# Israel’s rapid rollout of vaccinations for COVID-19

**DOI:** 10.1186/s13584-021-00440-6

**Published:** 2021-01-26

**Authors:** Bruce Rosen, Ruth Waitzberg, Avi Israeli

**Affiliations:** 1 Myers-JDC-Brookdale Institute, Jerusalem, Israel; 2 Hebrew University Paul Baerwald School of Social Work and Social Welfare, Jerusalem, Israel; 3 Department of Health Care Management, Faculty of Economics & Management, Technical University, Berlin, Germany; 4 Hebrew University Hadassah Medical School, Jerusalem, Israel; 5 Ministry of Health, Jerusalem, Israel

## Abstract

As of the end of 2020, the State of Israel, with a population of 9.3 million, had administered more COVID-19 vaccine doses than all countries aside from China, the US, and the UK. Moreover, Israel had administered almost 11.0 doses per 100 population, while the next highest rates were 3.5 (in Bahrain) and 1.4 (in the United Kingdom). All other countries had administered less than 1 dose per 100 population.

While Israel’s rollout of COVID-19 vaccinations was not problem-free, its initial phase had clearly been rapid and effective. A large number of factors contributed to this early success, and they can be divided into three major groups.

The first group of factors consists of long-standing characteristics of Israel which are extrinsic to health care. They include: Israel’s small size (in terms of both area and population), a relatively young population, relatively warm weather in December 2020, a centralized national system of government, and well-developed infrastructure for implementing prompt responses to large-scale national emergencies.

The second group of factors are also long-standing, but they are health-system specific. They include: the organizational, IT and logistical capacities of Israel’s community-based health care providers, the availability of a cadre of well-trained, salaried, community-based nurses who are directly employed by those providers, a tradition of effective cooperation between government, health plans, hospitals, and emergency care providers – particularly during national emergencies; and support tools and decisionmaking frameworks to support vaccination campaigns.

The third group consists of factors that are more recent and are specific to the COVID-19 vaccination effort. They include: the mobilization of special government funding for vaccine purchase and distribution, timely contracting for a large amount of vaccines relative to Israel’s population, the use of simple, clear and easily implementable criteria for determining who had priority for receiving vaccines in the early phases of the distribution process, a creative technical response that addressed the demanding cold storage requirements of the Pfizer-BioNTech COVID-19 vaccine, and well-tailored outreach efforts to encourage Israelis to sign up for vaccinations and then show up to get vaccinated.

While many of these facilitating factors are not unique to Israel, part of what made the Israeli rollout successful was its combination of facilitating factors (as opposed to each factor being unique separately) and the synergies it created among them. Moreover, some high-income countries (including the US, the UK, and Canada) are lacking several of these facilitating factors, apparently contributing to the slower pace of the rollout in those countries.

## Introduction

Worldwide, the year 2020 was dominated by the health and economic harm caused by the COVID-19 pandemic. That year ended with a glimmer of hope, as regulators began to approve COVID-19 vaccines and governments around the world began to administer them.

Table 1 presents data, by country, from the Our World in Data website [1] regarding the total number of doses administered and the number of doses administered per 100 population, as of the end of 2020. The table highlights two striking things about the State of Israel, whose end-of-year population was 9.3 million [2]. First, only three other countries (the US, China, and the UK) had administered more doses than Israel’s approximately 950,000. Second, Israel had administered almost 11.0 doses per 100 population, while the next highest rates were 3.5 (in Bahrain) and 1.4 (in the United Kingdom). All other countries had administered less than 1 dose per 100 population. As of the end of 2020, Israel’s rollout of COVID-19 vaccinations had clearly been rapid and effective.

**Table 1:** COVID-19 Vaccine Doses Administered as of the End of 2020

Listing the 10 countries reporting the most doses administered			
	Total	Doses per	Reporting
Country	Doses	100 pop’n	date
China	4,500,000	0.31	31-Dec
United States	2,794,588	0.84	30-Dec
United Kingdom	963,208	1.42	27-Dec
Israel	949,112	10.97	31-Dec
Germany	165,575	0.20	31-Dec
Canada	99,946	0.26	31-Dec
Bahrain	58,643	3.45	31-Dec
Russia	52,000	0.04	22-Dec
Poland	47,600	0.13	31-Dec
Mexico	24,998	0.02	30-Dec

Source: Our World in Data. https://ourworldindata.org/covid-vaccinations

Note: For each country, the table presents data for the latest date in December for which data were available in the database

As with any major accomplishment, a large number of factors contributed to Israel’s successful early rollout. This article begins with a brief overview of the Israeli rollout, and then discusses 12 factors that contributed to its early success, with the analysis focusing on the period until the end of 2020. The concluding remarks note limitations of the analysis and identify several avenues for further research.

## Overview of the Israeli rollout

Israel launched its COVID-19 vaccination campaign on December 20th, but preparations for it began months earlier. Over the course of 2020, Israel signed vaccine purchase contracts with several pharmaceutical companies at the forefront of COVID-19 vaccine development. By the time the US FDA had issued an emergency use authorization for the Pfizer-BioNTech COVID-19 vaccine^1^ on December 11, Israel already had contracts in place with Pfizer to purchase and receive a substantial (but undisclosed) number of doses of that vaccine by the end of December. Within days, and largely on the basis of the FDA authorization process, Israel’s Ministry of Health (MOH) followed with an authorization of its own.^2^,^3^

Israel’s MOH also determined (on December 16) that the initial target groups for vaccination would be people aged 60 and over, nursing home residents, other people at high risk due to serious medical conditions, and front-line health care workers [3]. The responsibility for vaccinating each of these groups was also clearly defined at that time:

- The primary responsibility for vaccinating the general population over age 60 and at-risk persons due to pre-existing medical conditions was assigned to Israel’s four competing non-profit health plans
- Responsibility for vaccinating nursing home residents was assigned primarily to Israel’s national medical emergency services organization - Magen David Adom (MDA).
- Responsibility for vaccinating front-line health workers was assigned to the hospitals and health plans with whom they work

**Fig. 1:**
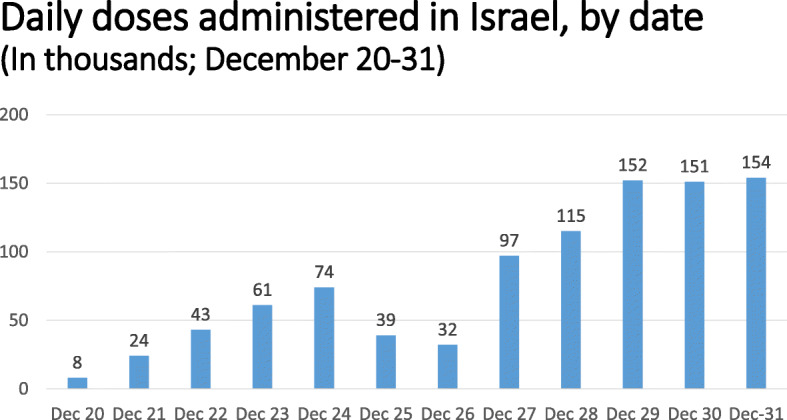
Daily doses administered in Israel, by date (In thousands; December 20–31). Source: Our World in Data. https://ourworldindata.org/covid-vaccinations

As indicated in Fig. 1, the number of people vaccinated per day began at approximately 8000 on December 20, quickly rose to over 70,000 by December 24, decreased over the following weekend, and then rose to over 150,000 by December 29.^4^ All of the vaccines administered in Israel during 2020 were those manufactured by Pfizer, and the vast majority of vaccines were administered by nurses.

At the same time, not all was well with the COVID-19 situation in Israel in December 2020, Israel – like many other countries – was experiencing a major increase in COVID-19 infections [4], including substantial morbidity among health care professionals. Thus, the vaccination campaign was launched at a very challenging time for Israeli health care.

In addition, the vaccination campaign experienced labor pains of its own. During the first few days of the rollout, it was quite difficult to schedule an appointment via the health plans’ call centers or digital tools. In some vaccination sites, not enough people in the target population showed up, and at the end of each day vaccines about to pass their expiration time had to be either thrown away or given to people not meeting criteria for first-round vaccinations. Even earlier in the day, some hospitals, health plans and other vaccine providers were somewhat lax about limiting vaccines to people meeting the official criteria, thereby increasing the total number of people vaccinated, but reducing the supply of vaccines available to the elderly and other at-risk groups. At some vaccination sites, family of health professionals and members of influential unions or occupations, were vaccinated even though they did not meet the criteria. And, while vaccination sites were set up throughout the country, including in the peripheral regions and in smaller villages and towns, the rate of vaccine uptake was markedly lower than average in Arab localities.

Moreover, as of December 2020 there were many uncertainties looking forward. There was lack of clarity about when the next vaccine shipments would arrive and how large they would be [5], leading to talk about a possible temporary suspension of first vaccinations (though Israel has been careful to set aside a second dose for all Israelis and foreign workers^5^ who received a first dose). It was also not clear what proportion of Israelis would ultimately sign-up for vaccinations, either due to general anti-Vax sentiments or to vaccine hesitancy specific to the new COVID-19 vaccines. In addition, it was not clear how the need to allocate nurses to the vaccination effort was affecting the delivery of other health services. And, as was the case worldwide, there continued to be uncertainties about how long the vaccine-conferred immunity would last, how effective it would be against new variants of the virus, and the extent to which it prevents transmission.

Still, there is no denying that, as of the end of 2020, Israel’s vaccination campaign had achieved a great deal – both in absolute terms and relative to other countries. Accordingly, despite its imperfections, and despite the uncertainties regarding how things would evolve in 2021, it is important to identify and analyze the factors that contributed to the success of Israel’s vaccine rollout in its initial phase.

## Selected factors contributing to Israel’s success

The specific factors contributing to Israel’s successful early rollout include, but are not limited to, the following:

1. Israel’s small size, in terms of both area and population, its relatively young population, and its relatively warm weather in December 2020
2. Israel’s centralized national system of government (as opposed to a federal system of government)
3. Israel’s experience in, and infrastructure for, planning and implementing prompt responses to large-scale national emergencies
4. The organizational, IT and logistic capacities of Israel’s community-based healthcare providers (the four health plans), which are all large and national in scope
5. The availability of a cadre of well-trained, salaried, community-based nurses who are employed directly by the health plans
6. The tradition of effective cooperation between government, health plans, hospitals, and emergency care providers – particularly during national emergencies – and the frameworks for facilitating that cooperation
7. The existence of well-functioning frameworks for making decisions about vaccinations and support tools for assisting in the implementation of vaccination campaigns
8. The rapid mobilization of special government funding for vaccine purchase and distribution
9. Timely contracting for a large amount of vaccines relative to Israel’s population
10. The use of simple, clear and easily implementable criteria for determining who had priority for receiving vaccines in the early phases of the distribution process
11. A creative technical response that addressed the demanding cold storage requirements of the Pfizer-BioNTech COVID-19 vaccine
12. Well-tailored outreach efforts to encourage the population to sign up for vaccinations

These specific factors can be divided into three major groups of factors, as follows:

A. Long-standing characteristics of Israel which are extrinsic to health care (items 1–3)
B. Long-standing characteristics of the Israeli health care system (items 4–7)
C. Specific actions taken as part of the COVID-19 vaccination effort (items 8–12)

We now provide additional information on each of the specific factors listed above, providing context and detail about the Israeli rollout not previously published in a comprehensive fashion. We also briefly cite examples of high-income countries in which those factors were not present, as vetted by country-specific experts who reviewed a draft of this article. We do so to demonstrate that many of these factors are neither trivial nor universal. We purposely do not present a comprehensive analysis of what have been the main influences on the speed of the rollout in any other country. Such comprehensive analyses are best carried out by experts writing about their own countries.

A. Long-standing characteristics of Israel which are extrinsic to health

1. Israel’s small size, in terms of both area and population, its relatively young population, and its relatively mild weather in December 2020

Israel has a population of 9.3 million. As a result, the number of doses required^6^ was a small fraction of the overall global supply, giving Israel agility and maneuverability in its purchasing. Meanwhile, Israel’s small size (about the same as New Jersey)^7^ and highly urbanized population, minimized the transport and storage challenges associated with the Pfizer-BioNTech COVID-19 vaccine (henceforth referred to as “the Pfizer vaccine”, for short). Inter alia, a single state-of-the-art medical warehouse sufficed to store the nation’s entire Pfizer vaccine reserve in the requisite ultra-low-temperature freezers.^8^ In addition, high population density increased the number of people who can easily access any particular community-based vaccination site – an important advantage with regard to the Pfizer vaccine.

Moreover, Israel’s population is relatively young (approximately 12% aged 65 or over) [6], reducing the amount of vaccine needed to rapidly vaccinate the bulk of the 60+ population.

And, with Israel being a small country geographically, the vast majority of aging and infirm Israelis apparently have a younger adult family member living in close geographic proximity,^9^ who can accompany them to a vaccination site, while providing moral support and transportation assistance. The relatively mild December in Israel in 2020 also made it easier for older people to get to vaccination sites.

These facilitating factors - Israel’s small size, a mild winter, and a relatively youthful population - did not exist in some high-income countries [8]. For example, the US has a population of over 300 million, greatly increasing the amount of vaccine needed by that country. Canada faced the challenges of a large geographic area and parts of the country in which the population density is low [9]; these created a need for more storage facilities, greater investment in transportation, and a need for many more vaccination sites.^10^ And several European countries have populations in which 20% or more are over age 65^11^ [10], meaning that vaccination coverage of the elderly would require a greater vaccine supply in terms of doses per population. Thus, many countries faced opening conditions – in terms of geography, population size, and age distribution - that were substantially more challenging than those faced by Israel.

2. Israel has a centralized national system of government^12^ (as opposed to a federal system of government)

Israel does not have states or regions which have independent decision-making authority on public health issues. While it does have active, and largely independent, local authorities and municipal governments, they play only a limited role in health care.^13^ As such, coordination of a public health response across different levels of government was not needed and this may have allowed the Israeli government more flexibility in designing its rollout. It also provided clarity in that the national government had the primary responsibility for the vaccination campaign, in terms of planning, financing, and implementation.^14^

In contrast, several high-income countries have federal systems, with significant implications for how public health efforts are organized. For example in the US, public health is administered and regulated primarily at the state level. On the other hand, it was the federal government that was responsible for promoting vaccine development, approving vaccines as safe and effective, procuring vaccines from pharmaceutical companies, and distributing them among, and to, states. This has led to some ambiguity regarding who is responsible and accountable for the success of the vaccination effort [11–14].

3. Israel has extensive experience in, and infrastructure for, planning and implementing prompt inter-sectoral responses to large-scale national emergencies.

Partly as a result of its challenging geo-political position, Israel has for many years invested substantially in preparing for large-scale emergencies, whether they be related to security, natural disasters, or health, based on an “all hazards” approach [15]. Inter-sectoral decision making bodies and implementation teams have been established, protocols have been developed, staff have been trained, and drills have been carried out. The scenarios for recent large-scale drills (pre-COVID-19) have included those in which large scale vaccination efforts had to be implemented. In Israel, the large community based health care providers – the health plans – are an integral part of national emergency preparedness drills. In addition, due to its security situation, Israel has amassed substantial real-world experience in responding to large-scale emergencies.

In the post-911 world, Israel is not the only country investing time, energy, and money in preparations for large-scale emergencies. But it is probably the case that few European and North American countries have as much experience as Israel does in responding to real-world emergencies and many of them apparently do not maintain surge capacity (relative to their size), to the extent that Israel does. Widespread public understanding that large-scale emergencies are not rare events may enhance the seriousness with which the public, professionals, and the government engage in emergency preparedness efforts [16]. Moreover, in many countries, community-based health care providers are not typically included in drills for large-scale emergencies.

In addition, Israel’s investment in emergency preparedness is supplemented by a culture of innovation and making rapid adjustments in response to changing circumstances.^15^

All of the above constituted important resources for Israel, as it planned and implemented the rollout of its vaccination campaign. It was quickly able to set up inter-sectoral frameworks for vaccine policy/program development and an inter-sectoral command center to oversee the implementation of the vaccination program.

B. Long-standing characteristics of the Israeli health care system

4. The organizational, IT and logistic capacities of Israel’s community-based providers (Israel’s four health plans), which are all large and national in scope^16^

Since 1995, Israel has had universal national health insurance coverage, financed primarily through income-related tax revenues. All permanent residents are free to choose from among Israel’s four large, competing, non-profit, health plans. These serve as Israel’s predominant, community-based health care providers. All four health plans are national in scope with primary care capabilities well-distributed across the country [18, 19]. They operate as well-run health care delivery systems based on national-regional-local hierarchies. All four health plans have a strong commitment to prevention as well as treatment, while most health plan members have long-standing relationships with, and a high level of trust in, their health plans [20, 21].

Moreover, all the health plans have well-developed electronic health records^17^ and strong capacities for electronic communications with their members [22]. Some of the health plans are also pioneers, internationally, in using their patient databases to carry out significant studies related to clinical care, epidemiology, and health policy [23–26].

The health plans are also have substantial experience in organizing and implementing nation-wide initiatives,^18^ large-scale mobilizations and emergency responses of various sorts [31]; this apparently contributed to their preparedness for the COVID-19 vaccination blitz. For example, the involvement of the health plans in periodic drills of responses to military bioterror exercises [32] have apparently honed their capacity to work with the IDF and other organizations in emergency situations. Experience of a complementary nature has been garnered over the past decade in the context of Israel’s National Program for Quality Indicators in Community Healthcare. That program monitors the performance of the health plans on approximately 70 quality measures; and each of the health plans undertakes large-scale, coordinated, organization-wide efforts to improve its performance on those measures [27].

In addition, the health plans’ annual influenza vaccination campaigns provided the health plans with experience in mobilizing staff for vaccinations as well as the rapid and efficient scheduling and processing of members for vaccinations. Moreover, in the months before the December 2020 COVID-19 vaccination campaign, the health plans carried out a particularly intensive flu vaccination campaign. This was motivated, in part, by concerns that the health system could get overwhelmed by the simultaneity of the COVD-19 pandemic and a major wave of influenza. As part of that influenza vaccination campaign, the health plans gained experience in renting out large public spaces as vaccination sites.

These long-standing capabilities have been vital to the central role that the health plans have played in the distribution and delivery of the Pfizer vaccine to their members. They were able to quickly distribute the vaccine to over 400 delivery points while meeting the challenging temperature and other logistic requirements of the Pfizer vaccine. The health plans were able to quickly and efficiently schedule hundreds of thousands of vaccination appointments for their members via call centers, apps, and organizational websites.

The health plans were able to quickly rent or otherwise access large facilities suitable for vaccinating large numbers of people.^19^ In some cases, the health plans outsourced part of their vaccination operations, building on substantial prior experience with outsourcing. They were able to staff the delivery effort in a quick and effective manner, operate the vaccine sites during extended work hours and throughout the week, with nurses, pharmacists, paramedics, administrators, and other health care professionals. Finally, the ongoing competition among the plans for members was an additional source of motivation (in addition to the dominant health protection objective) to excel in their vaccination efforts [33].

In contrast, most US and Canadian citizens^20^ are not enrolled in large-scale integrated delivery systems that have clear responsibility for their health and health care [28, 34], that can quickly organize and staff sites for large-scale vaccination efforts, and which can promptly address the logistical challenges involved in bringing together – in terms of time and place - the right mix of staff, patients, and vaccines.

The UK, despite having a single large-scale health care provider, the National Health Service, has also faced vaccine delivery challenges. One of those has been in clarity about which districts and which GPs were responsible for the health of each citizen.^21^ In the UK’s initial rollout, relative to the size of its population, and despite its larger geographic area, there were far fewer and often smaller sites for vaccine administration than in Israel. The UK relied on hospitals and relatively small, stand alone, or grouped, GP practices which seemed less suited than the one used by Israeli health plans to deliver a large-scale vaccination campaign [35].

In addition, in the UK, the vaccination strategy was implemented at the regional level (rather than by nationwide health plans). Gaps in organization and logistic capacities among regions may have compromised the efficiency and national consistency, of their vaccination campaigns [36].

5. The availability of a well-trained, salaried, cadre of community-based nurses who are directly employed by the health plans

In Israel, approximately one-third of the country’s nurses work in community settings, and about half of the community-based nurses work as salaried employees in Israel’s four non-profit health plans [37]. Many of those nurses have experience administering vaccinations, making it relatively easy for the plans to shift some of them from their regular tasks to the COVID-19 vaccination effort. These are skilled and well-trained professionals who could start vaccinating immediately.^22^ This was done despite Israel having a relatively low nurse to population ratio [38] and the prevalence of COVID-19 cases among nurses. It was accomplished via a large increase in nursing overtime hours and a partial deferral of other nursing tasks (e.g. monitoring of chronically ill patients). In addition, the health plans temporarily transferred some of the tasks which the nurses had been doing (e.g. phone calls to monitor the health of members who had become ill with COVID-19) to other health care professionals employed by the plans, such as social workers or speech therapists.^23^

Another key factor was that the scope of practice regulations for nurses authorizes them to independently assess which individuals meet the clinical criteria for vaccination, without requiring consultation with a physician or the physical presence of a physician.^24^

In addition to nurses employed by the health plans, supplemental staff were recruited from the Home Front Command of the Israel Defense Forces (IDF), private companies, and others. Israel also acted quickly to change the regulations governing the scope of practice of medics and para-medics so that they too could administer vaccinations.

Countries with mandatory health insurance systems typically face greater difficulties in staffing vaccination efforts. In Germany, for example, despite having health plans (currently 103 social health insurance funds), these plans do not have readily available staff to administer the vaccines.^25^ This is because the majority of health workers in the ambulatory sector, including primary care providers, are self-employed contractors of the plans rather than their employees (to whom they could more easily assign tasks). As a result, many of the vaccinations are being done by independent GPs, other physicians, medical assistants, retired health professionals, and others on a voluntary basis. They are paid by federal states, the federal government (via the liquidity reserve of the Central Reallocation Pool) as well as private health insurances and municipalities (not by health plans) [39, 40].

6. The tradition of effective cooperation between government, the defense forces, health plans, hospitals, emergency care providers, and local authorities – particularly during national emergencies – and the frameworks for facilitating that cooperation

While the various components of the health system are well versed in how to compete (and bicker) among themselves in ordinary times, they are equally versed in how to cooperate in cases of national emergencies and also on high-priority national objectives in ordinary times. In the current vaccination campaign, the Home Front Command of the Israel Defense Forces (IDF) is playing several vital roles. It is responsible for ultra-cold storage of the vaccines in a central location, transporting those vaccines to a large number of vaccination sites, and also organizing vaccination sites in small localities, which serve the members of all health plans. Local authorities also played an important role by making some of their very large facilities available as vaccination sties.

A related advantage enjoyed by Israel is that many of the leaders of the health system, and indeed of the government, have known one another personally for years, and in many cases have even worked together in the past. This is due, inter alia, to the country’s small size and the close ties forged during service in the IDF (particularly as officers in the IDF Medical Corps). These prior relationships facilitate communication and cooperation during national emergencies [41].

7. The existence of well-functioning frameworks for making decisions about vaccinations and support tools for assisting in the implementation of vaccination campaigns^26^

Israel has a well-established national Epidemic Management Team (EMT) and a well-established National Immunization Technical Advisory Group (NITAG) [42]. In the last quarter of 2020, the MOH general director appointed a special committee (“the COVID-19 vaccine prioritization committee”), comprised of members of those two long-standing committees, which then held regular (Zoom) meetings several times a week, and provided recommendations to the MoH.

In addition, Israel is one of the few countries that have a full population-based childhood immunization registry that includes all of the country’s children. Childhood vaccines are provided in Israel free of charge in all well-baby clinics in the country and doses are registered in a web-based registry [43]. Based on the experience from previous mass vaccination campaigns in Israel [44], the platform of the national registry was quickly adjusted and adapted to the current COVID-19 vaccine campaign. Notably residents in Israel all have a single unique identifier (ID) used in all health care facilities and allowing for ongoing timely data assembly on vaccine doses and number of vaccinees. The registry also allows follow up and assessment of post vaccination adverse events as well as providing real-world vaccine effectiveness data, i.e., “phase 4,” or post-marketing, data.

C. Specific actions taken as part of the COVID-19 vaccination effort

8. The rapid mobilization of special government funding for vaccine purchase and distribution

By mid-2020, senior Israeli policymakers, civil servants, and professionals realized that it was important for Israel to secure an adequate volume of vaccines as early as possible and promote their distribution and delivery. There was a consensus that this was important for Israel’s public health, economy, and perhaps for its security as well. There was also an early understanding that effective distribution and delivery would impose substantial additional costs on health care providers. Accordingly, the Ministry of Finance, in coordination with Israel’s political leadership, set aside substantial funds for vaccine acquisition, with an understanding that additional resources would have to be provided to support distribution and delivery.

Like Israel, many other high-income countries invested heavily in contracting with the pharmaceutical companies for future vaccine acquisition. Israel was also not alone in the allocation of special government funds to support vaccine delivery. However, in other countries such as the UK, the amount of such government support appears to have been limited. For example, in England many GP practices have reported that they are unable to participate in the vaccination program because the resources given to them (beyond the vaccine itself) were insufficient [45].

9. Timely contracting for a large amount of vaccines relative to Israel’s population

Over the course of 2020, Israel contracted with several of the pharmaceutical companies at the forefront of vaccine development.^27^ Not knowing which vaccines would prove to be effective, and when they would get regulatory authorizations, Israel distributed its risks by entering into advanced purchasing agreements (typically contingent on regulatory authorization) for more vaccine doses than it was projected to need.^28^

Details of these agreements, and of the deliberations and negotiations that preceded them, are not publicly available. However, it is likely that Israel’s ability to lock-in an early and adequate supply of vaccines was due to the combination of:

- Political leadership, including the active personal involvement of the prime minister,
- A willingness to pay premium (i.e. higher) prices [46],
- A highly professional purchasing division within the Ministry of Health [28], and
- An understanding on the part of the pharmaceutical companies that Israel has an unusually strong capacity to showcase the feasibility, and assess the impact of, a rapid rollout.

This last point may have been due, in part, to some pharma executives having had prior professional connections with Israel [47, 48].

This understanding of Israel’s potential as a lab and a showcase apparently also contributed to the collaboration agreement between Israel’s MOH and Pfizer (signed in early January), between Israel’s MOH and Pfizer, regarding the collection, sharing, and analysis of aggregate, real-world epidemiological data which can be used to assess when herd immunity is achieved [49, 50]. .This agreement appears to have played a role in expanding the supply of vaccines to Israel in January and beyond.

Israel secured a relatively large early supply of vaccines without having had produced any of those vaccines domestically. While Israel is working on a vaccine of its own, it was still in the testing stage at the end of 2020, so Israel’s early supply was fully imported.

Many (though not all) high-income countries, also entered into advanced purchasing contracts with multiple pharmaceutical companies prior to the regulatory authorizations of the vaccines. Some countries contracted for a quantity of vaccines well in excess of their populations [51]. At the same time, the “real-world epidemiological evidence collaboration agreement” between Pfizer and Israel appears to be somewhat unique.

Other aspects of the situation were different for most European countries. Procurement and purchase is done in coordination with the European Commission, in order to ensure a relatively similar share of vaccines among all countries [52]. Individual countries are not free to purchase as much vaccines as they want, even if they have sufficient funds to pay premium prices.

Moreover, in most countries, by the end of 2020, only a very small part of the contracted supply of vaccines had been delivered by pharmaceutical companies to governments. While exact data on this are unavailable, it appears that as of the FDA’s December 11 emergency use authorization of the Pfizer vaccine, the per capita vaccine supply was substantially greater in Israel than in most – if not all – other countries. Israel was also among the first countries to piggy-back onto the FDA’s authorization of the Pfizer vaccine with an authorization of its own.

10. The use of simple, broad, clear and easily implementable criteria for determining who had priority for receiving vaccines in the early phases of the distribution process

The initial target groups for vaccination in Israel were people aged 60 and over, nursing home residents, other people at very high risk due to serious specific medical conditions (mostly those related to compromised respiratory systems), and front-line health care workers. Each of these groups were well-defined. Moreover, the health plans, with their state-of-the-art EHRs were well-equipped to identify and reach out to members aged 60 or over and younger members with relevant medical conditions. Similarly, the Magen David Adom personnel had no problem identifying and accessing all nursing home residents, while hospitals and health plans could readily identify and contact the relevant employees and contracted workers. Together, these four groups constituted a relatively large number of people and a very high proportion of those most at risk of serious illness or death from COVID-19.

The primary objective of the initial prioritization scheme was to reduce mortality and severe illness related to CVOD-19, with special attention to the most vulnerable population groups. There was also an understanding that once the at-risk population is vaccinated, it will be easier to gradually open up the economy, without incurring major public health risks. Another objective of the prioritization was to ensure that the health care system not be overwhelmed, and this was advanced both by vaccinating the at-risk population and by vaccinating health care workers.

Before deciding to prioritize the groups at the greatest health risk, the relevant committee considered an alternative strategy – prioritizing those groups most involved in transmitting the disease. After deliberating the two strategies prioritization of those at greatest health risk emerged as the consensus. Primarily, this was due to the understanding that this would have the greatest contribution to population health. In addition, at the time there was a great deal of uncertainty about how many doses would be available for the initial rollout phase, and concern that there would not be enough to cover the relatively large groups involved in transmitting the disease.

Many other countries also have relatively clear and simple prioritization criteria, but due to limitations in the per capita vaccine supply their initial rollouts have focused on relatively narrow population segments, e.g. Austria [53], Denmark [54], and Spain [55, 56].

In the US the situation is more complex, as the CDC recommendations have called for “frontline essential workers” to be included in phase 1 of vaccination campaigns. It is clearly more difficult to determine which individuals are part of this group than it is to determine who is in a given age group.

11. A creative technical response to the demanding cold storage requirements of the Pfizer vaccine

According to Pfizer’s initial technical specifications, once the vaccine vials were removed from the ultra-low-temperature freezers there were to be transported to vaccination sites only in large trays containing 195 vials, enough for approximately 1000 doses. The professionals involved quickly realized that this requirement greatly limited the ability to carry out vaccinations in nursing homes, smaller localities, and other settings where the number of people in the top priority groups was well below 1000 [57]. With approval from Pfizer, “Israeli teams repacked the large ultra-frozen pallets into insulated boxes the size of small pizzas, allowing for distribution in smaller numbers and at more remote sites” [58]. This involved workers operating in very low-temperature refrigerators in which they did the repackaging [59].

Appendix presents additional information on the cold chain preservation efforts in Israel.

12. Well-tailored outreach efforts to encourage the population to sign up for vaccinations

A variety of steps were undertaken in Israel to promote public interest in vaccination [21, 60]. One key step was to wait until the highly respected US FDA had completed its rigorous review process before launching the vaccination campaign. The MOH, the health plans, and the hospitals then built on the FDA’s recognition of the vaccine as safe and effective, to launch a multi-pronged educational campaign to provide information, allay fears, and overcome hesitancy. The campaign made use of the mass media, social media, organizational websites, and more. An important part of this effort involved monitoring social media for anti-vax messages and addressing them head-on.

Another important step, taken by the health plans, was to create a number of different ways in which individuals could schedule a vaccination. These included by phone to a health plan call center, by computer via the health plan web site, or by mobile phone via a health plan app. In the initial days of the campaign there were technical difficulties in using these vehicles, which were overwhelmed by the surge in demand. However, these were subsequently sorted out and were widely used. This was facilitated by the relatively high proportion of Israelis who, even before the pandemic, had enrolled in their health plans’ secure portals for communicating personal information. These secure connections are also used to monitor and communicate side effects of the vaccine, providing individuals with a greater sense of security and thereby helping overcome vaccine hesitancy.

In addition, the mass media, in coordination with organizations involved in implementing the vaccine campaign, have provided daily updated on the number of Israelis vaccinated, accompanied by video clips and photos of large numbers of people getting vaccinated. These data and visuals may be providing reassurance to persons who might otherwise be hesitant to get vaccinated [61]. The thinking then becomes, “if so many Israelis are queueing up to get vaccinated voluntarily, then this is probably a good thing for me as well”. In addition, Israel’s position as the country with the most per capita doses administered has become a source of national pride, further encouraging Israelis to want to be part of this effort.

In addition, in Israel, the endorsements of cultural and intellectual leaders have been important in encouraging the general population to sign up for vaccinations. Key religious figures can make an important contribution to vaccine uptake rates among Ultra-Orthodox (Haredi) Jews and very devout Moslems. Accordingly, the Ministry of Health has invested substantial effort in recruiting the support of these religious leaders. Among the Haredi population, this bore fruit already in the first week of the vaccination campaign.

Among the Arab population, as of the end of 2020, additional outreach efforts were clearly needed. As indicated by Professor Nihaya Daoud, these should include opening additional vaccinations sites in Arab localities, operating mobile vaccination units that can reach small Arab villages, doing more to tailor the messaging and the media to the Arab population, having the health plans proactively schedule appointments by reaching out to Israel’s Arab citizens [62, 63].

The overall positive response in late December of persons age 60 and over to the opportunity to get vaccinated probably also reflected a strong and widespread desire to end their exposure to the substantial health and economic risks associated with the nearly year-long pandemic, as well as the limitations on day-to-day living imposed by the pandemic. It also probably reflects a reasonably high degree of trust in the safety and efficacy of the vaccines and the vaccination process. This, in turn, suggests that – at least on these matters – persons over 60 were trusting the government and other health system actors to act responsibly and in the public interest.

As of this writing, it is too soon to know how other countries will promote willingness in getting vaccinated among the general public, as many of them are just beginning to expand their vaccination campaigns beyond a narrowly defined target groups. Interestingly, in the UK people are individually invited to come in for a vaccination [64], rather than the Israeli approach where everyone meeting broad criteria has the opportunity to sign-up for a vaccination via health plan call centers, web sites, and apps. This can be seen as the difference between a pull approach and a push approach.

## Israel’s facilitating factors – an integrated view

Most of the factors discussed above are not unique to Israel. Perhaps the factor most specific to Israel is factor #4 - the organizational, IT and logistic capacities of Israel’s four large, national, health plans. These health plans are integrated health care systems which serve as the country’s predominant community-based health care providers. In very few other countries is the entire population enrolled in similarly effective delivery systems.

Aside from that, Israel’s relative success in the early rollout phase appears to be due to having a somewhat unique combination of factors (many of which, when taken individually, can be found in other countries) and taking advantage of potential synergies among those factors.

As Weintraub and colleagues have pointed out in a recent Health Affairs article, “The historical record suggests that to have a widely immunized population, leaders must invest in evidence-based vaccine delivery strategies that generate demand, allocate and distribute vaccines, and verify coverage” [65].

In Israel, the early availability of a relatively large per capita vaccine supply made it possible for Israel to give early priority to a large and clearly defined population group – persons over age 60. But administering vaccinations to roughly 10% of the population within 2 weeks required much more than having enough vaccine doses; it also required efficiently bringing together, at appropriate sites, vaccines, professionals to administer the vaccines, and people to receive those vaccines. In doing so, Israel built upon its emergency preparedness, inter-sectoral cooperation, and the organizational and technological capacities of the health plans. Similarly, it required a capacity to rapidly and flexibly mobilize a large number of health care professionals and to set up a sufficient number of sites which were organized for efficient throughput. The number and geographic distribution of vaccination sites was greatly enhanced by the creative technical response permitting the splitting-up of the large vaccination trays. Finally, to promote the interest of the population in getting vaccinated promptly, Israel built upon the long-standing trust relationships between individuals and their health plans by promoting a bandwagon effect and launching targeted outreach efforts.

Thus, in Israel, numerous facilitating factors were weaved together to enable it to effectively address the range of challenges noted by Weintraub.

## Concluding remarks

This analysis identified and discussed 12 factors which contributed to Israel’s relative rapid initial rollout of COVID-19 vaccinations. While many of these facilitating factors are not unique to Israel, there were important synergies among the factors that facilitated Israel’s initial rollout.

The analysis presented here is limited in several significant ways. The list of factors presented is intended to be illustrative, rather than comprehensive. Moreover, the discussions of some of the factors in the list just begin to scratch the surface on important issues. For example, it would be valuable to delve further into the factors that contributed to Israel’s ability to secure an early and adequate vaccine supply, and explore which of those factors were the dominant ones.

The analysis identified several features of Israeli health care which can lay the groundwork for a successful early rollout, without fully explaining how their potential was realized. For example, it is one thing to indicate that the health plans’ organizational and IT capacities were important assets; it is another to tell the fuller story of how these assets were marshalled effectively. Similarly, there is an important story to be told about how Israel built on an existing general receptivity to vaccines through a series of concrete and well-timed actions to address vaccine hesitancy and anti-vaxxer activity specific to the rapidly-developed and untraditional COVID-19 vaccines.

The analysis highlighted various features of Israeli health care that have made it a particularly good fit for the Pfizer vaccine, with its demanding temperature and transport requirements. Future research might consider whether Israel’s rollout would have been so much quicker than those of other countries if the first vaccine to receive FDA emergency use authorization had been that of Moderna or AstraZeneca, as the extreme cold storage requirements of the Pfizer vaccine were a major hurdle to rapid distribution in many countries.

Finally, our analysis ends at December 31, 2020. That year has ended, but the challenges countries face in vaccinating their populations continue. As of this writing, it is by no means clear whether, and to what extent, Israel will be able to sustain its early success in deploying COVID-19 vaccines. Much will depend on the extent to which Israel continues to build on its long-standing strengths and continues to adopt successful policies that are specific to the COVID-19 vaccination challenge.

Israel, like other countries, has faced, and will continue to face, numerous ethical issues in its vaccination campaign. Some, such as the priority to be given to undocumented workers, it has in common with other countries. Others, such as the nature and scope of assistance to be given to the Palestinian Authority, are unique to Israel. In the weeks and months ahead, it will be important for these issues to be discussed seriously – in the Knesset, in the news media, and in academic articles.

The rollout has, of course, continued to evolve beyond the end of 2020. In the process of that evolution, it has been possible to extend immunization to additional groups, such as teachers and persons under age 60, and significantly increase the percentage of the population covered (including among the Ultra-Orthodox and Israeli Arabs). New issues have arisen, including the questions of how to vaccinate the home-bound, and what priority should be given to prisoners. In the future, it will be important to supplement this analysis of the rollout’s initial phase with analyses of how it evolves in 2021.

## Data Availability

Not applicable.

## Appendix

### Transport and cold storage in Israel of Pfizer COVID-19 vaccines^29^

The Pfizer vaccines are brought to Israel via air transport in specially designed, temperature-controlled containers/ utilizing dry ice to maintain the recommended storage temperature conditions of − 70 °C. The thermal shippers are then transported by trucks from the airport to a state-of-the-art medical warehouse operated by S.L.E, where they are stored in ultra-low-temperature freezers. From the S.L.E warehouses, vaccines are towed and transported in good distribution practice (GDP) trucks to hospitals, clinics, designated immunization centers, and additional points of use (POUs). GDP conditions include temperature validated containers and monitored, temperature-controlled, trucks with GPS enabled devices. The vaccines arrive to the point of vaccination in trays containing 195 vials (in certain circumstances this amount may be reduced according to clinical/logistic needs). After towing, the vaccines expire in 5 days, when refrigerated at temperatures of 2–8 °C.

At the vaccination point, the vaccines are transferred from the validated container and stored in temperature controlled validated refrigerators to maintain 2–8 °C. The refrigerators are monitored constantly and are connected to a central alert system that tracks and records deviations in the storage temperature. Once the vials have been removed from these refrigerators, they are stable for 2 h at room temperature. For preparation of vaccine the vials are reconstituted with normal saline (0.9%NaCl); at point they must be used within 6 h.

In early phase, when providers had to work with platters of 95 vials, they used mostly large public spaces which can accommodate many people (e.g. The Arena in Jerusalem). Once a method was developed to split up the platters, in agreement with Pfizer, the vaccination activity increasingly took place in the large number of community clinics that are spread throughout Israel.

## Abbreviations

CBS: Central Bureau of Statistics
IDF: Israel Defense Forces
IJHPR: Israel Journal of Health Policy Research
MDA: Magen David Adom
MOH: Ministry of Health
POU: Point of use
UK: United Kingdom

## Footnotes

1 The formal name of the vaccine is: Pfizer-BioNTech COVID-19 vaccine, BNT162b2 (brand name “Comirnaty”)

2 Personal communication, Boaz Lev, January 2021.

3 At this stage, the vaccine has been authorized for use by people aged 16 and over. There is no prohibition against vaccinating a pregnant woman or a woman planning a pregnancy. A person with an acute illness, including a fever of 38 degrees Celsius or above, will not be vaccinated until they have recovered from that illness. According to current guidelines, the following are not authorized to receive the vaccine: people under age 16, people with a history of severe allergic reaction (who should contact the warehouse nurse to find out if they can be vaccinated), and people who were confirmed as having been ill with COVID-19.

4 Israel did not face the issue, present in some other countries, of large numbers of healthcare workers being on vacation during the week between Christmas and New Year’s Day.

5 Foreign workers carrying for frail elderly living at home or in nursing homes were included among the health care workers who were vaccinated in the first phase of the rollout.

6 Israel’s vaccination program covers all persons covered by Israel’s National Health Insurance Law as well as additional groups, such as soldiers (who are entitled to health care via the IDF) and prisoners (who are entitled to health care via the Prisons Law). Undocumented migrant workers are not covered. The Palestinian Authority is responsible for the health and health care of Palestinian residents of Gaza and the West Bank. This includes responsibility for providing vaccinations against COVID-19. Thus, in general, they are not included as part of Israel’s vaccination program. In contrast, Palestinian workers legally residing in Israel by virtue of a residence permit granted to them as part of a family reunification procedure, as they are covered by the NHI.

7 We recognize that several European countries have areas similar to that of Israel. The intention here is to distinguish this characteristics of Israel’s area primarily from those of the US, China, Canada and other large-area countries.

8 To some extent, these freezers were on hand prior to the pandemic. Others were purchased during 2020 in preparation for the expected need to store large quantities of the Pfizer vaccine.

9 According to the 2009 Social Survey carried out by Israel Central Bureau of Statistics, 79% of the elderly in Israel met at least once a week with a family member who does not live with them in the same household [7]. The comparable figure in the 2019 Social Survey was 77%.

10 Confirmed by Greg Marchildon, personal communication, January 2021.

11 These include 23% in Italy, 22% in Germany, and 20% in France

12 Sometimes referred to as a unitary system of government

13 Nonetheless, some of these local authorities made an important contribution to the vaccination campaign by making large municipal facilities available as vaccination sites.

14 Of course, even with a single level of government involved, there were issues to be resolved about the distribution of responsibility between various agencies of the national government, such as the Ministry of Health, the IDF, the Knesset, the Prime Minister, and the Cabinet. In the case of the vaccine campaign, these issues seem to have been resolved effectively. This has not been the case regarding other aspects of the pandemic response.

15 This has been touted as one of the main factors that have contributed to Israel’s Startup Nation status [17].

16 The health plans, which are financed primarily by government via a capitation formula, are responsible for ensuring that their members receive all needed services included in a government-determined benefits package.

17 An individual’s electronic health record can be accessed by all physicians working with the individual’s health plan, thereby promoting continuity of care. Moreover, if that individual is hospitalized, the hospital can also access key elements of his/her EHR (such as underlying health conditions and relevant community-based treatments), the individual’s primary care physician is automatically notified about the hospitalization, and key data about the care in the hospital are shared with the health plan electronically.

18 This is true for efforts to improve ongoing care, such as nation-wide, collaborative systems for monitoring and improving quality [27, 28]. It is also true for efforts to address new challenges, such as the rise in obesity [29] and efforts to arrange health insurance for undocumented migrants [30].

19 In the early days of the vaccination campaign, a large proportion of the vaccinations took place in large, rented facilities. Once Israel received approval from Pfizer to split up the large trays of vaccine vials, an increasing proportion of the vaccinations shifted to community-based clinics.

20 While Canada has a national health insurance system, most of its hospitals and physicians operate independently, rather than as part of large, integrated systems of care.

21 The information presented in this paragraph has been confirmed by Fiona Sim, personal communication, January 2021.

22 Similarly, in hospitals, nurses are salaried employees and they too were rapidly mobilized for the vaccination campaign.

23 David Mossinson, personal communication, January 2021.

24 At the same time, individuals with certain sensitivities were referred by the nurses to physicians for further evaluation.

25 The information presented in this paragraph has been confirmed by Juiane Winkelman, via personal communication, 2021.

26 The authors are indebted to Chen Stein-Zamir for contributing this section of the article.

27 The Israeli government has promised the pharmaceutical companies (Pfizer and Moderna), the health plans, and the public that the vaccines for COVID-19 will be included within purview of the Insurance for Victims of Vaccines Law, 1989. This means that the government will compensate any individuals whose health was harmed as a result of receiving the vaccine.

28 Israel did not make a separate acquisition of vaccines for the IDF. The vaccines acquired are being used for civilians and military (as well as police) personnel in an integrated fashion. Soldiers were not listed as a distinct target population for the first phase of the vaccine rollout.
